# Whole-Genome Sequences of Three Psychrotolerant *Mycolicibacterium* sp. Strains Isolated from Antarctic Soil

**DOI:** 10.1128/mra.00123-23

**Published:** 2023-05-30

**Authors:** Sho Shimada, Kotaro Aoki, Ryosuke Nakai, Shota Miura, Kohji Komori, Sakae Kudoh, Satoshi Imura, Yoshikazu Ishii, Kazuhiro Tateda

**Affiliations:** a Department of Microbiology and Infectious Diseases, Toho University School of Medicine, Tokyo, Japan; b Department of Respiratory Medicine, Tokyo Medical and Dental University (TMDU), Tokyo, Japan; c Bioproduction Research Institute, National Institute of Advanced Industrial Science and Technology (AIST), Hokkaido, Japan; d National Institute of Polar Research, Research Organization of Information and Systems, Tokyo, Japan; e Department of Polar Science, School of Multidisciplinary Sciences, SOKENDAI (The Graduate University for Advanced Studies), Tokyo, Japan; University of Southern California

## Abstract

We report the whole-genome sequences of three psychrotolerant *Mycolicibacterium* strains, TUM20983, TUM20984, and TUM20985, isolated from Antarctic soils. Taxonomic analyses indicate that these strains are putative new species. These genome sequences may provide insight into the cold adaptation mechanisms of *Mycolicibacterium* spp. through future comparative genomic studies.

## ANNOUNCEMENT

Mycobacterial species are distributed throughout natural environments; however, limited information is available for species living in Antarctica (1–3). In this study, we report the whole-genome sequences (WGSs) of three psychrotolerant *Mycolicibacterium* sp. strains isolated from Langhovde and Skallen ice-free areas in East Antarctica.

Surface soil samples were collected in January 2019 and preincubated as previously described (4). Subsequently, supernatants of soil suspensions were incubated on Reasoner’s 2A (R2A) agar plates (Becton, Dickinson, Franklin Lakes, NJ) at 15°C. DNA was extracted from each colony using the QIAamp DNA minikit (Qiagen, Hilden, Germany). Nearly full-length 16S rRNA gene sequences were obtained by PCR using the primer pair 27F and 1525R (5), followed by Sanger sequencing. Each sequence was subjected to a BLASTn search against the NCBI nucleotide/nonredundant (nt/nr) database. One strain isolated from a sample from Langhovde (69.2404 S, 39.7655 E) and two from a sample from Skallen (69.6044 S, 39.7458 E) were related to members of the family *Mycobacteriaceae* and were further analyzed by WGS. These strains were all confirmed to grow at 4°C to 25°C and failed to grow at temperatures higher than 30°C.

Genomic DNA (gDNA) for both short- and long-read WGS was prepared from the same colony that appeared 7 days after incubation at 15°C on R2A agar plates. gDNA was extracted using method 5 described by Bouso and Planet (6). Paired-end (300-bp) short-read sequencing was conducted using a MiSeq instrument (Illumina, San Diego, CA, USA), after preparation using the Illumina DNA prep kit and the MiSeq reagent kit v3 (Illumina). Long-read sequencing libraries were prepared using the rapid barcoding kit (Oxford Nanopore Technologies, Oxford, UK) and sequenced using the R9.4 flow cell on a MinION sequencer (Oxford Nanopore Technologies), with no size selection. The generated short and long reads were quality filtered and adapter trimmed using the Trimmomatic v0.39 (quality score cutoff, 20) (7) and NanoFilt v2.8.0 (quality score cutoff, 6; minimum length, 5,000 bp) (8), respectively. Using a combination of short and long reads, we generated hybrid *de novo* assemblies with Unicycler v0.5.0 (9). The contigs were polished three times using Pilon v1.24 (10). The genomes of the three strains were annotated using the DDBJ Fast Annotation and Submission Tool (DFAST) v1.2.18, incorporating quality assessment using average nucleotide identity (ANI) (11). Default parameters were used for all software unless otherwise noted.

The basic genomic properties of the three strains are summarized in Table 1. These strains shared identical 16S rRNA gene sequences and high ANI values (98.9% to 99.2%), calculated using FastANI v1.3 (12). However, the strains showed low 16S rRNA gene identity to sequences deposited in the NCBI nt/nr database by BLASTn search (top hit: Mycobacterium sp. strain AT1; GenBank accession number KY649294.1; identity, 98.22%) and low ANI values with genomes in the type strain genome database used in the taxonomy check of DFAST (closest match: *Mycolicibacterium arabiense* JCM 18538^T^; GCA_010731815.2; ANI value, 80.72%). These results indicate that the three strains represent putative new *Mycolicibacterium* species. These genome sequences may provide insights into the low-temperature adaptation and survival strategies of *Mycolicibacterium* species.

**TABLE 1:** Basic properties of the genomes of three *Mycolicibacterium* strains

Strain ID	Sequencing data					Genome statistics							ANI (%) with:		
	Instrument	No. of reads	*N*_50_ (bp)	Total no. of bases (bp)	Depth (×)	Length (bp)	No. of scaffolds	*N*_50_ (bp)	G+C content (%)	No. of CDSs^*a*^	No. of rRNAs	No. of tRNAs	TUM20983	TUM20984	TUM20985
TUM20983	MiSeq	1,752,414	350	526,736,763	90.3	5,836,029	3	5,771,957	67.5	5,690	6	47			
	MinION	36,432	11,973	394,968,054	67.7										
TUM20984	MiSeq	1,871,574	350	562,605,786	93.6	6,008,954	2	3,093,446	67.3	5,857	6	48	98.941		
	MinION	5,135	12,250	56,562,806	9.4										
TUM20985	MiSeq	2,230,088	350	670,378,463	110.7	6,054,104	1	6,054,104	67.3	5,841	6	47	98.984	99.226	
	MinION	23,782	11,308	247,199,909	40.8										

a CDSs, coding DNA sequences.

### Data availability.

The WGSs of TUM20983, TUM20984, and TUM20985 were deposited at DDBJ/EMBL/GenBank under the accession numbers BSPY01000001 to BSPY01000003, BSPZ01000001 and BSPZ01000002, and AP027291, respectively. The raw sequence reads are available in the Sequence Read Archive under the accession numbers DRR439532, DRR439534, and DRR439536 (MiSeq reads) and DRR439533, DRR439535, and DRR439537 (MinION reads), respectively.
